# *Arabidopsis* genes, *AtNPR1, AtTGA2* and *AtPR-5*, confer partial resistance to soybean cyst nematode (*Heterodera glycines*) when overexpressed in transgenic soybean roots

**DOI:** 10.1186/1471-2229-14-96

**Published:** 2014-04-16

**Authors:** Benjamin F Matthews, Hunter Beard, Eric Brewer, Sara Kabir, Margaret H MacDonald, Reham M Youssef

**Affiliations:** 1 United States Department of Agriculture, Agricultural Research Service, Soybean Genomics and Improvement Laboratory, Beltsville, MD 20705, USA; 2 Fayoum University, Fayoum, Egypt

**Keywords:** Arabidopsis, Composite plants, Gene overexpression, Jasmonic acid, Resistance, Salicylic acid, Soybean, Soybean cyst nematode, Transgenic roots

## Abstract

**Background:**

Extensive studies using the model system *Arabidopsis thaliana* to elucidate plant defense signaling and pathway networks indicate that salicylic acid (SA) is the key hormone triggering the plant defense response against biotrophic and hemi-biotrophic pathogens, while jasmonic acid (JA) and derivatives are critical to the defense response against necrotrophic pathogens. Several reports demonstrate that SA limits nematode reproduction.

**Results:**

Here we translate knowledge gained from studies using *Arabidopsis* to soybean. The ability of thirty-one *Arabidopsis* genes encoding important components of SA and JA synthesis and signaling in conferring resistance to soybean cyst nematode (SCN: *Heterodera glycines*) are investigated. We demonstrate that overexpression of three of thirty-one *Arabidoposis* genes in transgenic soybean roots of composite plants decreased the number of cysts formed by SCN to less than 50% of those found on control roots, namely A*tNPR1*(33%), *AtTGA2* (38%), and *AtPR-5* (38%). Three additional *Arabidopsis* genes decreased the number of SCN cysts by 40% or more: *AtACBP3* (53% of the control value), *AtACD2* (55%), and *AtCM-3* (57%). Other genes having less or no effect included *AtEDS5* (77%), *AtNDR1* (82%), *AtEDS1* (107%), and *AtPR-1* (80%), as compared to control. Overexpression of *AtDND1* greatly increased susceptibility as indicated by a large increase in the number of SCN cysts (175% of control).

**Conclusions:**

Knowledge of the pathogen defense system gained from studies of the model system, *Arabidopsis,* can be *directly* translated to soybean through direct overexpression of *Arabidopsis* genes. When the genes, *AtNPR1, AtGA2,* and *AtPR-5,* encoding specific components involved in SA regulation, synthesis, and signaling, are overexpressed in soybean roots, resistance to SCN is enhanced. This demonstrates functional compatibility of some *Arabidopsis* genes with soybean and identifies genes that may be used to engineer resistance to nematodes.

## Background

Plant parasitic nematodes cause billions of dollars in losses each year worldwide [1-3]. The root-knot nematode, genus *Meloidogyne*, attacks over 3000 species of plants [4,5], while the soybean cyst nematode (*Heterodera glycines*) has a much narrower host range and is responsible for almost one billion dollars per year in losses in the US [2,3]. Although some soybean genotypes are resistant to specific populations of SCN, no soybean genotype is known to be resistant to all SCN populations. Several genes conferring partial **R**esistance to ***H****eterodera****g****lycines* (Rhg) have been mapped, and, recently, genes at the rhg1 and Rhg4 loci have been elucidated [6-9].The defense response of soybean to SCN has been examined at the physiological, genetic, and molecular level, and several reports indicate that salicylic acid (SA), jasmonic acid (JA), ethylene (ET), and indole acetic acid (IAA) play a role in resistance and susceptibility of plants to nematodes [8,10-15]. However, the roles of defense-related hormones and specific components of their synthesis and signaling pathways in providing resistance in plants to nematodes are unknown.

The plant defense response is complex. Plants launch a myriad of local and systemic defense responses to protect themselves from invasion by pests and pathogens. Several hormones are involved in inducing the defense response and regulating defense response networks, including SA, JA, ET, and IAA [11,12,16-18]. Plants react to pathogen-associated or microbe–associated molecular patterns (PAMPs/MAMPs) which are sensed by plants through leucine-rich repeat (LRR) receptors. PAMPs signal stomatal closure and stimulate innate immunity in plants. In general, SA activates the defense response to biotrophic and hemi-biotrophic pathogens, induces systemic acquired resistance (SAR), and triggers the expression of SAR-associated pathogenesis related genes *PR-1*, *PR-5*, and others [12,18]. The role of specific components of SA regulation, synthesis, and signaling in defending plants against parasitic nematodes is not well understood. However, SA does play a role in decreasing susceptibility to root-knot nematode (RKN) in cow pea [19] and tomato [20,21], and to the cyst nematode, *Heterodera schachtii,* in *Arabidopsis*[10]. Likewise, JA also plays a role in resistance of plants to nematodes. Foliar spraying of tomato with JA reduced galling and the final population of RKN (*M. incognita*); [22-26], as did pre-treatment of tomato seeds with JA [21], indicating a role for JA in plant resistance to nematodes.

Little is known of the role of specific components of SA regulation and signaling in the interaction of soybean with the soybean cyst nematode (SCN; *Heterodera glycines*), the major pest of soybean in the US. Although soybean genes conferring resistance to SCN have been identified, these do not provide resistance to all SCN populations. Resistance in soybean to SCN is multigenic, and several major loci for resistance have been identified [15,27-31]. For example, in soybean cv Peking, several genes (*rhg1*, *rhg2*, *rhg3*, and *Rhg4*) have been reported that confer resistance to SCN race 1 [15,32], yet none of these genes confers complete resistance to all SCN populations. Therefore, we are applying to soybean a portion of the vast knowledge that has been gained from studies on the model plant *Arabidopsis* and its large array of mutants on the role of SA and JA in the plant defense response to identify important components that may be useful in decreasing susceptibility of plants to nematodes, and especially of soybean to SCN.

**Figure 1:**
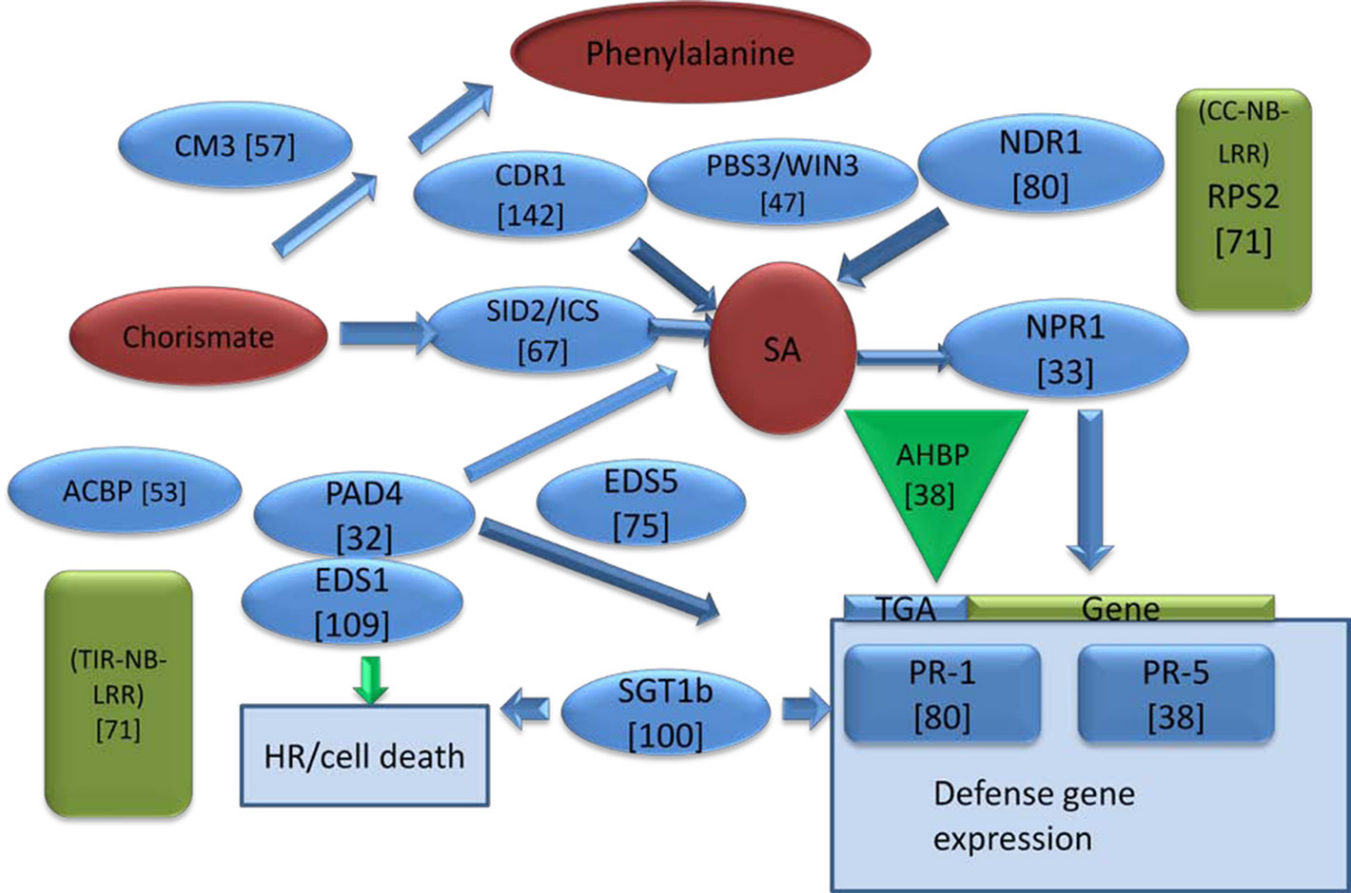
**Representation of some components involved in regulation and biosynthesis of salicylic acid and associated signaling.** SCN Female Indices (FI) of the genes examined are provided in brackets. Control Female Index = 100.

*Arabidopsis* has been used widely as a model system to study plant defense pathways, usually with bacterial and fungal pathogens [11,12,17,33-39]. Much attention has been focused on SA production and signaling pathways using *Arabidopsis* mutants infected with bacterial and fungal pathogens (Figure 1) [12,16-18,35-44]. For example, when a virulent strain of the biotrophic pathogen, *Pseudomonas syringae*, attacks *Arabidopsis*, the AvrRPS4 effector protein of *P. syringae* secreted by the type III secretion system is detected by the plant receptor RPS4, a Toll-interleukin-nucleotide binding-leucine-rich repeat (TIR-NB-LRR) that mediates the induction of antimicrobial defenses to provide disease resistance. The nucleo-cytoplasmic protein ENHANCED DISEASE SUSCEPTIBILITY 1 (EDS1), which is a lipase-like protein, connects RPS4 with downstream defense pathways and regulates the accumulation of SA [45,46]. EDS1 is essential for production of the hypersensitive response and mobilization of defense pathways [47-49]. EDS1 can dimerize and interact with another lipase-like protein, phytoalexin deficient 4 (PAD4) [48,49]. Both *EDS1* and *PAD4* are required for the accumulation of SA and they are involved in ROS signaling [46,50]. PAD4 protein is required for amplification of weak signals resulting from pathogen infection. Another important component of the defense response is EDS5, a multi-drug transporter member of the MATE family of transporters [50].

It is postulated that SA can be synthesized through two different pathways in *Arabidopsis*[51,52]. One pathway involves the enzyme isochorismate synthase (ICS; EC 5.4.4.2), which catalyzes the conversion of chorismate to isochorismate. The enzyme chorismate mutase (CM; EC 5.4.99.5) catalyzes the competing chemical reaction and converts chorismate to prephenate. This would divert chorismate to produce other compounds, such as phenylalanine and tyrosine. The *Arabidopsis sid2-2* (*SA INDUCTION-DEFICIENT 2)* mutation has been mapped to the locus encompassing the *ICS* (*SID1*) gene [52]. Upon synthesis, SA can bind directly with NPR1, which is encoded by *AtNPR1* (*NONEXPRESSOR OF PR1*), also known as NIM1 (NON-INDUCIBLE IMMUNITY 1). NPR1 is an SA receptor that is a transcriptional regulator of genes involved in the SA-dependent defense response [53], including the SA marker gene *PR-1* (*PATHOGENESIS RELATED 1*). NPR1 interacts with transcription factor TGA2 family members, including AHBP-1b, and the complex binds to SA-responsive promoter elements of *PR-1* and other SA-dependent defense genes to regulate expression [54].

SA can also be regulated independently of EDS1 and PAD4. NDR1 (NON-RACE SPECIFIC DISEASE RESISTANCE 1) is a positive regulator of SA that works independently of EDS1 and PAD4 [55] NDR1 is an integrin-like protein that can associate with RIN4, while RIN4 can associate with RPM1 and RPS2 [56]. NDR1 may play a role in electrolyte release upon infection of *Arabidopsis* by *P. syringae*, while RIN4 regulates stomatal apertures in conjunction with H + −ATPases of the plasma membrane of *Arabidopsis* during pathogen attack [57].

**Figure 2:**
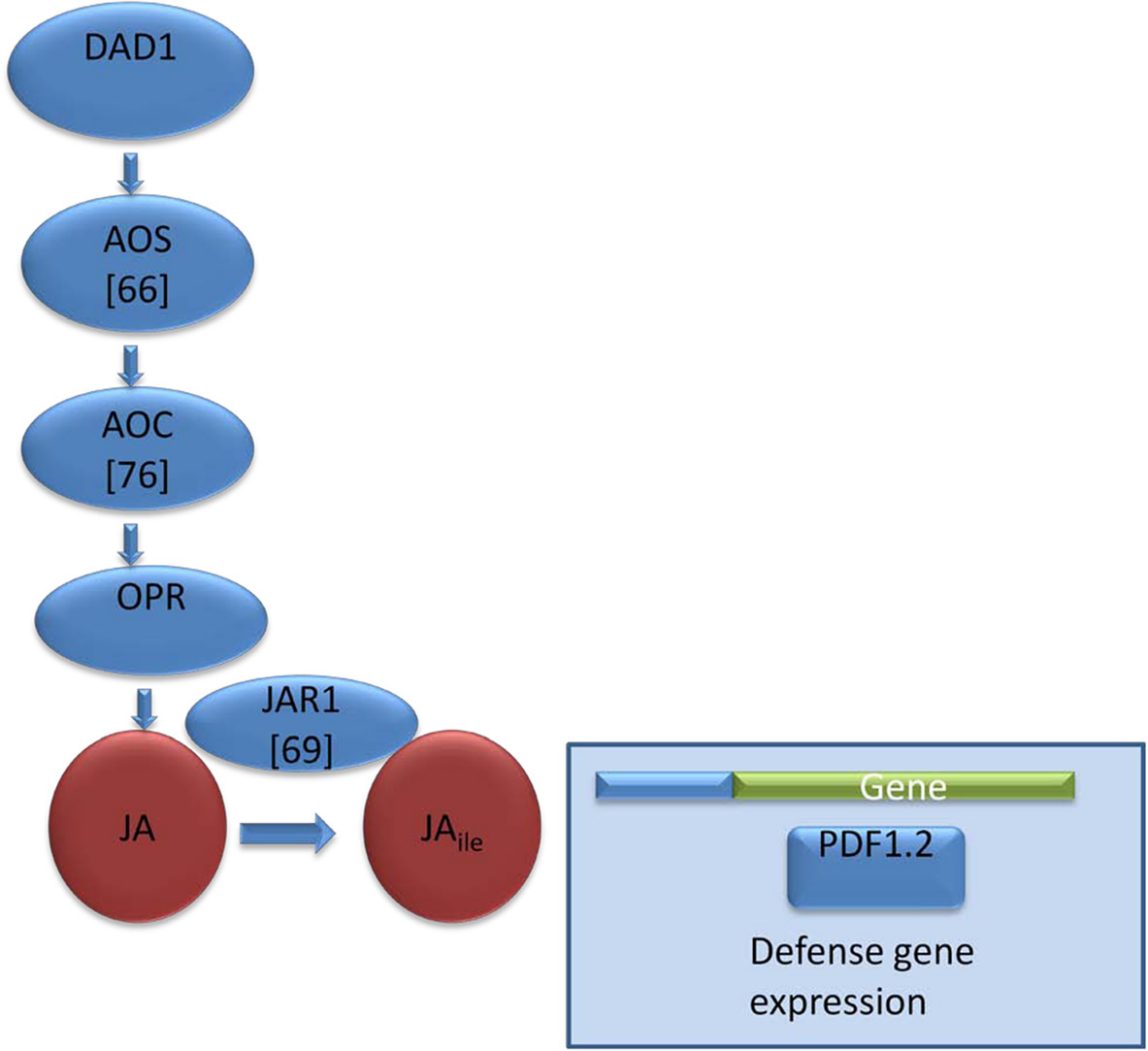
**Representation of some components involved in regulation and biosynthesis of jasmonic acid and associated signaling.** The SCN FI is provided in brackets.

JA, JA_ile_, and related lipid-derived compounds also act as signals in the plant defense response and are associated with resistance to necrotrophic pathogens [16,58,59]. The pathway leading to JA and JA_ile_ synthesis and some components related to JA signaling and control are depicted in Figure 2. Allene oxide synthase (AOS) and allene oxide cyclase (AOC) are two enzymes important to JA synthesis. JAR1 encodes an ATP-dependent JA-amido synthase that conjugates isoleucine with JA to form JA_ile_, which plays an essential role in JA signaling.

In this paper, we examine the role of some components of the plant defense response in conferring resistance in soybean to SCN. We show that specific *Arabidopsis* genes, namely A*tNPR1*, *AtTGA2, AtICS1*, and *AtPR5* which encode components of SA regulation, biosynthesis, and downstream effectors, can decrease susceptibility of soybean to SCN when expressed in transgenic soybean roots. We also demonstrate that expression of *Arabidopsis* genes encoding AOS, AOC, and JAR1, which are involved in the synthesis and modification of JA, only modestly decrease susceptibility of soybean roots to SCN. These results indicated that some *Arabidopsis* genes can be directly used in soybean, thus directly applying knowledge of the defense response gained from studies using *Arabidopsis* as a model system to soybean to decrease susceptibility to nematodes.

## Results

### Expression of *Arabidopsis* genes in soybean roots

SA and JA are well known regulators of the plant defense response as described through studies of the model plant, *Arabidopsis*. To determine if some of these *Arabidopsis* genes could be directly used in soybean to translate knowledge from *Arabidopsis* to soybean, we selected and cloned genes encoding numerous components of SA and JA synthesis, regulation, and signaling from the literature describing the defense response of *Arabidopsis* to pathogens (Table 1). To broaden the scope of our study, we also selected several *Arabidopsis* genes less well defined in function or that represented other portions of the plant defense response less dependent upon SA and JA. Open reading frames (ORFs) of thirty-one *Arabidopsis* genes were cloned into the gene expression vector pRAP15 [8,60,61] using the primers listed in Additional file 1: Table S1. The inserted ORFs were sequenced to confirm their identity and to ensure their sequence was conserved. The vector with insert was transformed by *Agrobacterium rhizogenes* K599 into cells at the base of the cut stem of soybean seedlings. Approximately 35 days after transformation, untransformed roots were removed from the composite plants, and the transformed roots were inoculated with SCN.

**Table 1:** The effect of expression of thirty-one Arabidopsis genes on the number of SCN cysts at 35 dai was determined The number of plants (n), Standard Error of the Mean (SEM) and Female Index (FI) are provided. The control FI = 100

**Phytozome ID**	**Gene**	**Gene**	**SEM**	**pRAP15**	**SEM**	**FI**	**P-value**
		**n**		**n**		**(% of control)**	
AT1G64280.1	NPR1	12	16	10	26	33	0.003
AT5G06950.1	TGA2	9	13	27	13	38	<0.0001
AT1G75040.1	PR-5	10	13	10	26	38	0.001
AT4G24230.6	ACBP3	12	11	15	21	53	0.03
At4G37000.1	ACD2	11	10	15	21	55	0.04
AT1G69370.1	CM3	11	22	10	26	57	0.03
AT5G42650.1	AOS	12	11	15	21	66	0.11
AT5G50260.1	CEP1	12	11	15	21	66	0.11
AT1G74710.2	ICS1	12	13	28	10	67	0.002
AT2G46370.4	JAR1	12	7	24	6	69	0.07
AT3G03600.1	RPS2	9	16	15	21	71	0.22
AT5G48485.1	DIR1	10	14	15	21	72	0.22
AT4G39030.1	EDS5	13	16	11	26	75	0.39
AT3G25760.1	AOC	9	17	28	10	76	0.06
AT3G26830.1	PAD3	14	4	17	9	79	0.28
AT5G54250.1	DND2	11	15	15	21	79	0.35
AT3G20600.1	NDR1	10	13	11	26	80	0.46
AT2G14610.1	PR-1	8	23	28	10	80	0.20
AT5G13160.1	PBS1	11	19	27	13	81	0.20
AT1G02170.1	LOL3	11	13	28	10	83	0.10
AT3G25070.1	RIN4	10	27	10	19	89	0.53
AT4G20380.8	LSD1	7	31	28	10	95	0.78
AT4G11260.1	SGT1b	16	7	17	9	100	0.99
AT5G64930.1	CPR5	12	7	17	9	102	0.97
AT1G12560.1	ExPA7	12	5	14	6	108	0.72
AT3G48090.1	EDS1	11	22	11	26	109	0.80
AT2G17265.1	DMR1	11	20	15	21	110	0.67
AT1G05180.1	AXR1	15	17	17	9	115	0.67
AT4G21610.1	MC2	10	27	28	10	135	0.06
AT5G33340.1	CDR1	12	23	11	26	142	0.23
AT5G15410.1	DND1	13	16	17	9	175	0.047

The effect of expression of thirty-one genes on the number of SCN cysts at 35 dai (days after inoculation) was determined (Table 1). Six genes decreased the number of cysts more than 40%, thus conferring partial resistance to SCN. Three of these genes, *AtNPR1, AtTGA2,* and *AtPR-5,* decreased the number of cysts more than 60%, while three others, *AtACBP3, AtACD2,* and *AtCM3* decreased the number of cysts 40%. One Arabidopsis gene, *AtDND1,* increased the number of cysts of SCN to 175% of the control, thus making the soybean roots more susceptible to SCN (Table 1).

**Figure 3:**
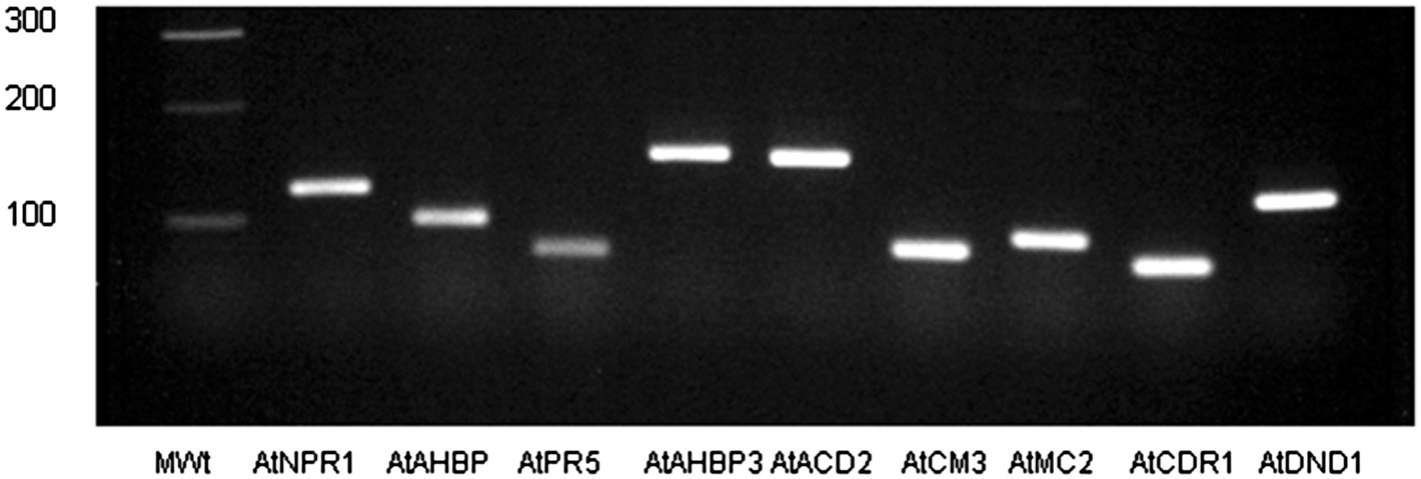
**Expression of transcripts from each gene in transformed roots.** RNA was converted to cDNA and used as template for PCR amplification of a fragment of each gene. Agarose gel containing amplicons representing a portion of *AtNPR1, AtTGA2, AtPR-5, AtACBP3, AtACD2, AtCM-3, AtMC2, AtCDR1, AtDND1*, respectively. Molecular weight markers (MWt) are shown in the first lane. Each lane represents a transgenic root from an individual plant. The cDNA from RNA extracted from wild type (WT) roots did not produce an amplicon for any of these genes. However, cDNA from the wild type was present, and an amplicon was produced by PCR when the cDNA was used as template with primers for a soybean control gene. RNA was extracted from three roots, individually, and independently made into cDNA. Each was examined by PCR and visualized as above. All samples from transgenic roots produced amplicons according to the appropriate *Arabidopsis* gene.

RNA was extracted from a subset of transformed roots for genes listed in Additional file 2: Table S2 to check for expression of the *Arabidopsis* gene by PCR. The amplicons were separated and visualized by gel electrophoresis and staining (Figure 3) to confirm that the ORFs were expressed in the composite root. All roots tested expressed the transcript.

**Figure 4:**
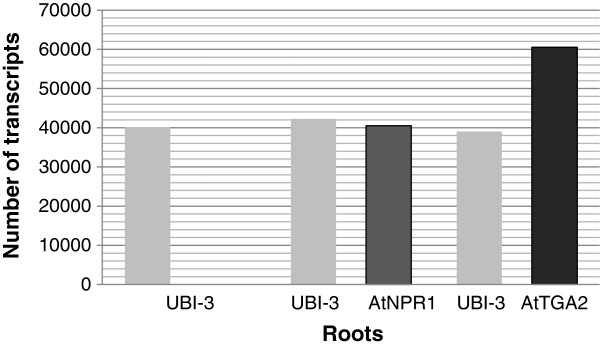
**Number of transcripts of *AtNPR1 *and *AtTGA2 *in transformed roots were determined in roots transformed with *AtNPR1 *and *AtTGA2.*** No transcripts of either gene were detectable in the control roots. In all samples, the expression level was similar for the soybean housekeeping gene encoding ubiquitin-3 (*GmUBI-3*).

In addition, the abundance of transcript of two genes providing the most protection against SCN, *AtNPR1* and *AtTGA2,* was determined by qRT-PCR using gene specific primers (Additional file 2: Table S2). Transcript number was calculated using the sigmoidal method [62]. The number of transcripts of *AtNPR1* was 40,500 molecules and for *AtTGA2* was 60,500 molecules in transformed roots, while no transcripts of either gene were detectable in the control roots. In all samples, the expression level was similar for the housekeeping gene encoding ubiquitin-3 (Figure 4).

**Figure 5:**
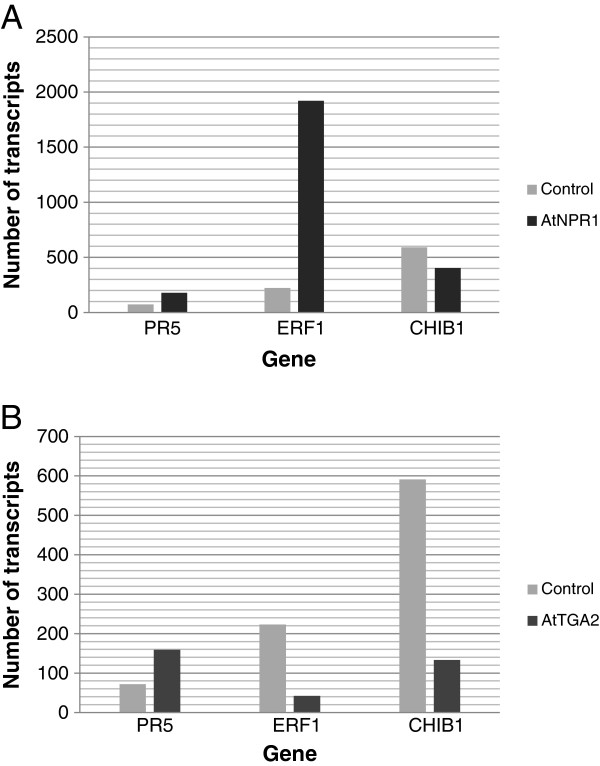
**The number of transcripts of three defense-related genes, *GmPR5*, *GmCHIB1*, and *GmERF1 *** **was determined in roots transformed with (A) *AtNPR1 *** **and (B) *AtTGA2 *** **using qRT-PCR.**

The number of transcripts of three defense-related genes, *GmPR5*, *GmCHIB1*, and *GmERF1*was also determined using qRT-PCR (Figure 5). In roots overexpressing *AtNPR1,* there were 178 transcripts of *GmPR5*, while in roots overexpressing *AtTGA2,* there were 159 transcripts. In control roots, there were only 38 transcripts of *GmPR5*. Thus, GmPR5 was elevated approximately 4-fold in roots overexpressing *AtTGA2.* Transcripts of *GmCHIB1* were also elevated in these roots. There were 403 transcripts of *GmCHIB1* in roots overexpressing *AtNPR1* and 133 transcripts in roots overexpressing *AtTGA2.* There were only 53 transcripts of *GmCHIB1* in control roots. This represents an increase in expression of *GmCHIB1* by approximately 8- and 2.5-fold in soybean roots overexpressing *AtNPR1* and *AtTGA2,* respectively. In contrasts, the number of transcripts of *GmERF1* decreased in soybean roots overexpressing *AtNPR1.* Only 42 transcripts of *GmERF1* were present, whereas control plants contained 1921 transcripts. Similarly, roots overexpressing *AtTGA2* contained fewer *GmEFR1* transcripts than in control roots with only 75 transcripts present. Thus, *GmERF1* expression was greatly decreased in both sets of transgenic roots.

### SA-related genes

Activation of the defense response in plants is initiated through several parallel signaling pathways. In gene-for-gene resistance, host resistance (R) proteins indirectly recognize pathogen effectors to initiate resistance [63]. The coiled-coiled-nucleotide-binding site-leucine-rich repeat (CC-NB-LRR) and the TIR-NB-LRR classes of proteins are two major sub-groups of R protein [64]. In this study, we selected the Arabidopsis protein NON-RACE-SPECIFIC DISEASE RESISTANCE 1 (NDR1) as a representative CC-NB-LRR R-protein, because of its known role in activating the SA-mediated defense response in Arabidopsis*.* RPS2, encoded by *AtRPS2*, was selected as a representative of the TIR-NB-LRR class of proteins. Both *AtNDR1* and *AtRPS2* were overexpressed in transgenic soybean roots to determine their effect on SCN growth and maturation as measured by the female index (FI), which expresses the number of mature SCN females 35 days after root inoculation as a percent of the control value. Overexpression of AtNDR1 slightly inhibited SCN development (FI = 80), while AtRPS2 had a slightly greater inhibitory effect (FI = 71), but the effect was not statistically significant (P > 0.05) (Table 1) for either gene.

Arabidopsis NON-EXPRESSOR OF PATHOGENESIS-RELATED GENES 1 (AtNPR1) is downstream of the R proteins NDR1 and RPS2. NPR1 is a key regulator of SAR and plays a critical role as a SA signal transducer in *Arabidopsis*[38,44]. When *AtNPR1* was overexpressed, the FI decreased to 33% of the control. *AtNPR1* had the lowest FI value among the *Arabidopsis* genes tested in this study (Table 1).

**Figure 6:**
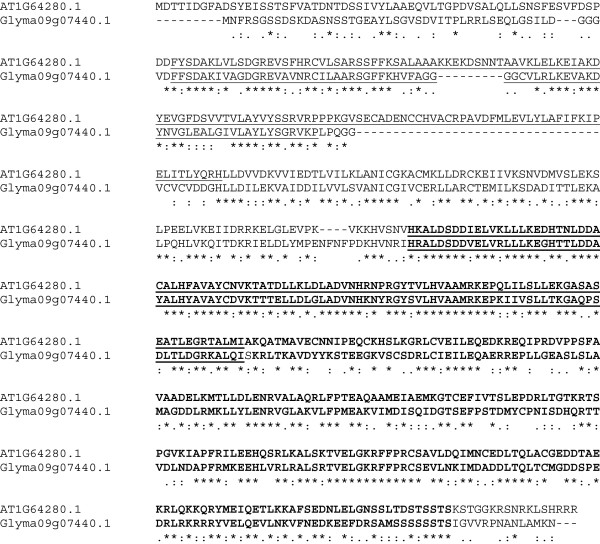
**Alignment of the *Arabidopsis *and soybean protein sequences of NPR1 using Clustal 2.1, showing the BTB/POZ domain (underlined), ankyrin repeats (domain CLO465; underlined and bold)), and NPR1/NIM1-like defense protein C terminal (bold).** (*) = identical aa; (:) = highly conserved aa substitution; (.) = conserved substitution.

Alignment of the 593 aa of AtNPR1 with its closest soybean counterpart, the product of Glyma09g07440.1, indicates conservation of only 273 aa, although there are also many conservative substitutions (Figure 6). There are five soybean genes encoding proteins closely related to AtNPR1. The protein encoded by Glyma09g07440.1 is most closely related to the protein encoded by Glyma09g02430.1, and these are closely related to the proteins encoded by Glyma15g13320.1, Glyma14g03510.1, and Glyma02g45260.1. All five soybean putative NPR1 proteins contain a BTB/POZ domain, ankyrin repeats (domain CLO465), a NPR1/NIM1-like defense protein C terminal, and a domain of unknown function (DUF3420), as does AtNPR1.

**Figure 7:**
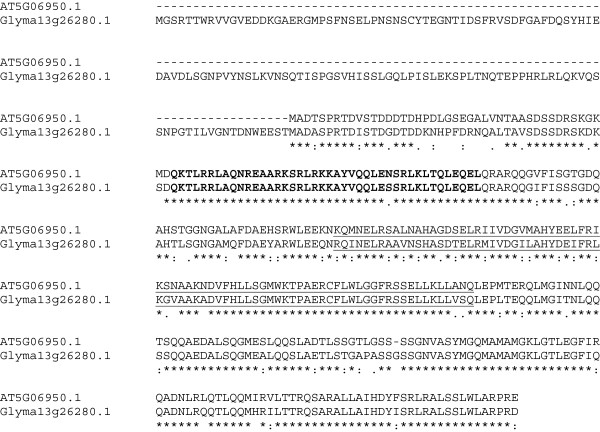
**Multiple sequence alignment of the *Arabidopsis *and soybean protein sequences of TGA2-1B using Clustal 2.1, showing the bZIP domain (bold) and the DOG1 domain (underlined).** (*) = identical aa; (:) = highly conserved aa substitution; (.) = conserved substitution.

Expression of *AtTGA2*, encoding the TGA-type basic leucine zipper bZip transcription factor AHBP-1B, in soybean roots decreased the FI of SCN to 38% of the control (Table 1). There are numerous soybean homologs of *AtTGA2*. The four most highly conserved are Glyma13g26280.1, Glyma15g37220.1, Glyma20g39050.2, and Glyma10g44270.1. The amino acid sequence of AtTGA2 is closely related to Glyma20g24766.1 (5e-56; Figure 7). The alternative transcript, Glyma20g24766.2, is exactly the same, except it lacks the 5′ leader sequence. The Glyma20g24766.1 transcript appears to contain a chloroplast transit sequence as predicted using ChloroP1.1, Technical University of Denmark, http://www.cbs.dtu.dk/services/ChloroP/, while Glyma20g24766.2 does not contain this 5′ transit sequence. We used the alternate sequence Glyma20g39050.2 that lacks a 5′ leader sequence that is also missing in AtTGA2. The AtTGA2 and GmTGA2 protein sequences are highly conserved in the bZIP domain [65-68], with only two aa differences in the 39 aa domain. This region is highly conserved with only eight aa substitutions in the 65 aa region. A second, domain, DOG1 [69] is found toward the carboxy terminus and is involved in the control of seed dormancy [54]. The DOG1 domain is less conserved between these Arabidopsis and soybean proteins.

**Figure 8:**
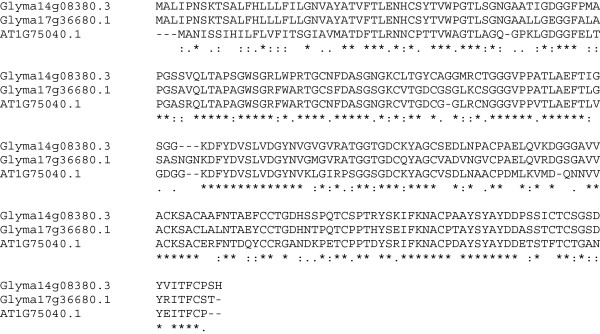
**Multiple sequence alignment of the *Arabidopsis *and soybean protein sequences of PR-5 using Clustal 2.1.** (*) = identical aa; (:) = highly conserved aa substitution; (.) = conserved substitution.

In *Arabidopsis*, SA interacts with the receptor NPR1, which then interacts with the transcription factor TGA2 to modulate the transcription of some genes, including *PR-1* and *PR-5*. Thus, we examined the effect of overexpression of *AtPR-1* and *AtPR-*5 on the FI of SCN. Overexpression of *AtPR-1* did not have a statistically significant effect (FI = 80; P = 0.2) on the number of SCN cysts. In contrast, when *AtPR-5* was overexpressed in soybean roots, the FI was decreased to 38% of the control (Table 1), while overexpression of *AtPR-1* had only a mild effect on the FI, which was 80% of the control. In soybean there is a large family of over 15 *Gm-PR-5* genes with Glyma14g08380 and Glyma17g36680 being the most closely related to the *Arabidopsis PR-5* gene, AT1G75040 (Figure 8). The sequences of the proteins encoded by the *Arabidopsis* and two most closely related soybean genes are highly conserved.

Three well-studied genes involved in SA are AtPAD4, AtEDS1, and AtEDS5. Previously, we demonstrated that expression of *AtPAD4* greatly decreased the development of female SCN to 32% of the control [60]. Here, we examined the effect of overexpression of AtEDS5 and AtEDS1, neither of which significantly affected SCN development, with FI values of 77 and 109, respectively.

SA can be synthesized through a shorter pathway involving ICS, or through a longer pathway through phenylalanine using CM. Therefore, we expressed *AtICS1* and *AtCM-3* in soybean roots to determine their effects on SCN maturation. Overexpression of *AtICS1* in roots had a modest inhibitory effect (FI = 67, P = 0.002). Because *AtICS1* did not strongly affect the FI, we anticipated that expression of CM would have minimal inhibitory effect on the FI of SCN or, perhaps, increase susceptibility, because CM competes with ICS for the common substrate chorismate. However, expression of *AtCM-3* also significantly inhibited SCN growth (FI = 57, P = 0.03).

WIN3, encoded by *HOPW1-1-INTERACTING 3* (*WIN3*), is involved in regulating SA and disease resistance [70-72], though the mechanism is unclear [73]. Overexpression of *AtWIN3* decreased the FI of SCN to 47% of the control.

*Arabidopsis* ACBP3 is an acyl-coenzyme A (CoA)-binding protein [74]. Transgenic *Arabidopsis* overexpressing *AtACBP3* displayed constitutive expression of the pathogenesis-related genes *PR-1* (unknown function), *PR-2* (β-1,3-glucanase)*,* and *PR-5* (osmotin), and resistance to *P. syringae* DC3000 was dependent upon the NPR1 mediated signaling pathway [75]. Overexpression of *AtACBP3* in soybean roots resulted in a decrease of the FI of SCN to 53% of the control.

Overexpression of *AtCPR5* (*CONSTITUTIVE EXPRESSOR OF PATHOGEN RELATED GENES 5*) in soybean roots had little effect on the female index of SCN. *CPR5* mutants constitutively express PR genes at a high level [76,77]; display defects in cell division, endoreduplication, and cell wall production [78,79]; and are of reduced stature and exhibit the formation of spontaneous lesions [79,80].

### JA-related genes

JA and related compounds are important in defense responses, especially the response to necrotrophic pathogens [81,82]. JA and JA_ile_ are synthesized through a series of enzymatic steps (Figure 2), including the enzymes allene oxide synthase (AOS (DDE2); EC 4.2.1.92); allene oxide cyclase (AOC; EC 5.3.99.6); and jasmonic acid-amido synthetase (JAR1; EC 6.3.2.-.). JAR1 conjugates JA with isoleucine to form JA-Ile, which is considered to be one of the active forms of JA [74-77]. Overexpression of the *Arabidopsis* genes *AtAOS, AtAOC*, and *AtJAR1* did not influence the FI of SCN in a statistically significant manner (66% (P = 0.11), 76% (P = 0.06), and 69% (P = 0.07) of the control, respectively; Table 1). These data do not suggest overexpression of [83] these genes will improve resistance in soybean to SCN.

### Other *Arabidopsis* genes

Overexpression of *AtRIN4* genes in soybean roots had little effect on the female index of SCN (Table 1). RIN4 is a negative regulator of innate immunity in plants [57]. It regulates stomatal closure. It appears to be peripheral to the defense response of soybean roots to nematode attack, as it did not significantly alter the FI of SCN.

In *Arabidopsis,* the chloroplast protein ACCELERATED CELL DEATH 2 (ACD2) modulates the amount of cell death that occurs in *Arabidopsis* leaves infected with *P. syringae*[84]. When the *AtACD2* gene was overexpressed in soybean roots, the FI of SCN was reduced to 55% of the control (Table 1).

Cysteine endopeptidases containing a C-terminal endoplasmic reticulum retention signal, KDEL, are involved plant cell death [85]. Overexpression of the cysteine endopeptidase encoded by *AtCEP1* reduced the FI of SCN to 66% of the control which was not statistically significant (Table 1).

### Arabidopsis genes that increased susceptibility when overexpressed

**Figure 9:**
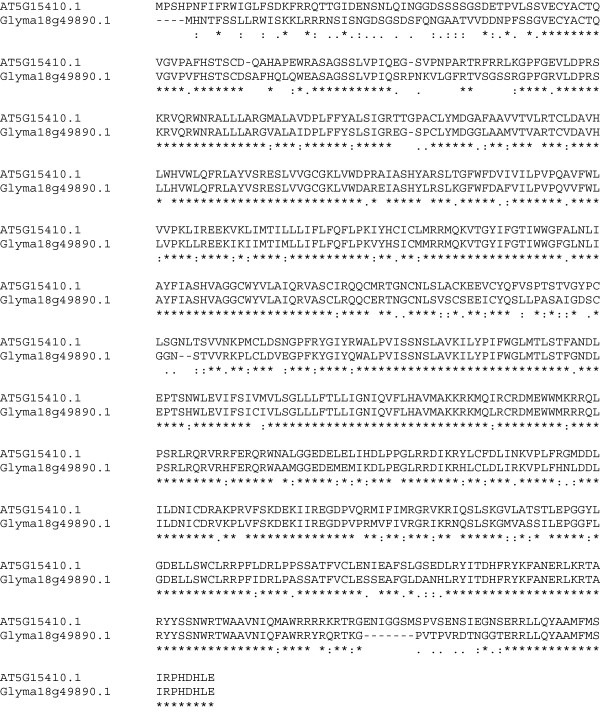
**Multiple sequence alignment of the *Arabidopsis *and soybean protein sequences of DND1 using Clustal 2.1.** (*) = identical aa; (:) = highly conserved aa substitution; (.) = conserved substitution.

The *AtDND1* gene AT5G15410.1 encodes the cyclic nucleotide-gated cation channel protein DEFENSE NO DEATH 1 (DND1), and is involved in production of the hypersensitive response [86]. The *Arabidopsis dnd1* mutant produces elevated amounts of SA. Overproduction of *AtDND1* in soybean roots did not provide resistance to SCN; rather, it enhanced susceptibility. The FI of transgenic soybean roots containing *AtDND1* was 175% of the control, the largest increase in susceptibility of the genes tested here (Table 1). The protein sequence of DND1 is highly conserved between *Arabidopsis* and soybean (Glyma18g49890.1) as indicated in Figure 9. It contains a cyclic nucleotide-binding domain as indicated by a significant (e-value = 5.8) Pfam-A match.

Overexpression of two other *Arabidopsis* genes did not alter susceptibility of soybean to SCN at a statistically significant level. The first gene, *CONSTITUTIVE DISEASE RESISTANCE 1* (*AtCDR1*), encodes an aspartic protease [87]. When *AtCDR1* was overexpressed in soybean roots, the FI was 142% of the control (P = 0.23) (Table 1). The second gene *AtMC2* (*LOL2* (*LSD1-LIKE*)) encodes the positive regulator of cell death during the hypersensitive response and is a conserved paralog of LSD1 [88,89]. LSD1 is a negative regulator of plant programmed cell death. Overexpression of *AtMC2* in soybean roots yielded a FI of 135% (P = 0.06) (Table 1).

## Discussion

Resistance to SCN is a multigenic trait and several genetic loci have been mapped [15,27-29,90-93]. Recently, the identity of genes residing at the *rhg1* and *Rhg4* loci have been reported [6-9] which confer some resistance to SCN. However, none of these loci alone provides full resistance to any one SCN population. For example, in a cross between soybean cv Essex and Forrest, *rhg1* and *Rhg4* accounted for about 65% of the variation in resistance found in the resultant inbred population to SCN [94]. Other soybean genes have been identified that confer partial resistance to SCN when overexpressed in roots [8,9,60,61].

An option to developing resistance to nematodes is to use defense-related genes that have been described in the literature. Much of the literature describing work with the defense response of *Arabidopsis* is concerned with elucidating defense response signaling, regulation, and pathways important to bacterial and fungal pathogens that attack the leaf of the plant. Although this research may be applicable to resistance of plants to nematodes and to agronomic crops such as soybean, little published work has yet translated the knowledge gained from these important studies in *Arabidopsis* to soybean and other important crops. Direct translation of research in *Arabidopsis,* includes transforming *Arabidopsis* genes directly into crop plants to determine if they have a positive or negative effect in that crop on disease resistance. Here we have shown that some *Arabidopsis* genes, when overexpressed in soybean roots, are compatible and confer resistance to SCN.

SA plays an important role in the plant defense response to pathogens. SA regulates SAR, local disease resistance, host cell death, and expression of genes involved in the defense response [44]. In tomato, SA is important to resistance to three RKN species [21]. Transgenic tomato expressing *NahG,* encoding salicylate hydrolase which degrades SA, was less resistant to RKN. However, resistance to RKN induced in tomato through the application of cell suspensions of the biocontrol bacterium *Pseudomonas aeruginosa* is independent of the accumulation of SA [95]. Thus, it may be that SA plays a role in providing resistance to RKN in tomato, but there may be other SA-independent mechanisms that also confer resistance. Uehara et al. [96] showed that inhibition of the SA signaling pathway in tomato harboring the *Hero A* gene increased susceptibility to *Globodera rostochiensis.* A protective effect against gall eelworm was seen in tomatoes when seeds were soaked in SA [14]. These and other reports show a strong link between SA and nematode resistance.

Examination of *Arabidopsis* mutants has played a key role in our understanding of the defense response and is the subject of many reviews [17,18,35,36,38,39]. It is postulated that SA can be synthesized through two different biochemical pathways [52,97]. In the first pathway, chorismate is converted to isochorismate via the action of ICS; then, SA is produced from isochorismate by isochorismate pyruvate lyase. Examination of *ICS1* mutants, *sid2-1* and *sid2-2,* of *Arabidopsis* indicate that loss of ICS1 activity dramatically decreases SA levels [48]. Most SA synthesized and relevant to plant defense in Arabidopsis appears to be made through this pathway. The *sid2* mutant does not accumulate SA upon inoculation with *P. syringae*, and *PR-1* expression is reduced greatly. However, *PR-2* and *PR-5* are expressed [90]. The second possible pathway diverts chorismate via CM to phenylalanine, which is converted to cinnamic acid by phenylalanine ammonia lyase (PAL) and progresses through a series of reactions to form SA. Previously, we demonstrated that overexpression in transgenic soybean roots of two different soybean genes encoding PAL did not greatly affect SCN maturation, with FI values of 94 and 111% [8]. However, here we show that overexpression of CM and ICS, representatives of the two different pathways, decrease the FI to 57% and 67% of the control, respectively. However, overexpression of PAL, CM, or ICS alone does not confer resistance to the level provided by overexpression of several other SA-related genes individually.

### Genes decreasing susceptibility of soybean to SCN

If the SA-related defense response is a major factor in soybean resistance to SCN, then components regulated by SA may confer resistance to SCN when overexpressed. Our results show that several *Arabidopsis* genes involved in SA regulation, synthesis, and signaling conferred resistance to SCN when overexpressed in soybean roots. The *Arabidopsis* genes *AtNPR1, AtTGA2, AtPR-5,* and several others related to SA strongly decreased the FI of SCN in transgenic soybean roots (Figure 1). NPR1 is a master regulator of the SA-related defense response, and it is a receptor for SA [51]. NPR1 binds SA and interacts with TGA transcription factors, such as the transcription factor AHBP-1b/TGA2, perhaps through its ankyrin domain [54]. NPR1 and TGA transcription factors work downstream of SA and are important to the expression of the genes encoding PR-1, PR-5, and others [98-102]. Expression of these genes is completely abolished in *Arabidopsis* plants carrying the *npr1* mutation [83]. Recently, Pant et al. [103] demonstrated that a Gm ortholog, Glyma09g02430, of *Arabidopsis* NPR1 reduced SCN cysts to approximately 30% of the control, in agreement with our data for overexpression of *AtNPR* (FI = 33%)*.* There are reports in numerous plants indicating that overexpression of NPR1 results in defense against fungal and bacterial pathogens. Overexpression of *AtNPR1* in *Arabidopsis* conferred resistance to *P. syringae* and *Peronospora parasitica*[104]. Overexpression of *AtNPR1* in rice conferred resistance to the rice bacterial blight pathogen *Xanthomonas oryzae*[105]. Overexpression of *AtNPR1* in wheat conferred resistance to fusarium head blight, caused by *Fusarium graminearum.* The apple *MpNPR1* gene confers resistance to two fungal pathogens of apple, *Venturia inaequalis* and *Gymnosporangium juniper-virginianae*[106,107] complemented *Arabidopsis npr1-1* mutants with soybean homologs *GmNPR1-1* and *NPR1-2,* and PR-1 was induced in the transformed plants after infection with *P. syringae* and after treatment with the SAR inducer, 2,6-dichloroisonicotinic acid.

NPR1 interacts with the transcription factor TGA2 to modulate expression of some plant defense genes, such as PR-1 and PR5 [108]. When we overexpressed AtTGA2*,* the FI of SCN was decreased to 38, showing that TGA2 can also confer resistance to SCN. This is further supported by our data showing the reduction of the FI to 38% of the control due to PR-5 overexpression. PR-5 is a thaumatin-like protein involved in the defense response [109], perhaps creating transmembrane pores to disrupt the membranes of pathogens [110]. Some PR-5 proteins exhibit anti-fungal activity [109,111]. When *Prunus domestica* PR-5 was overexpressed in transgenic *Arabidopsis,* the plants displayed more resistance to the fungal pathogen *Alternaria brassicola*[112].

**Figure 10:**
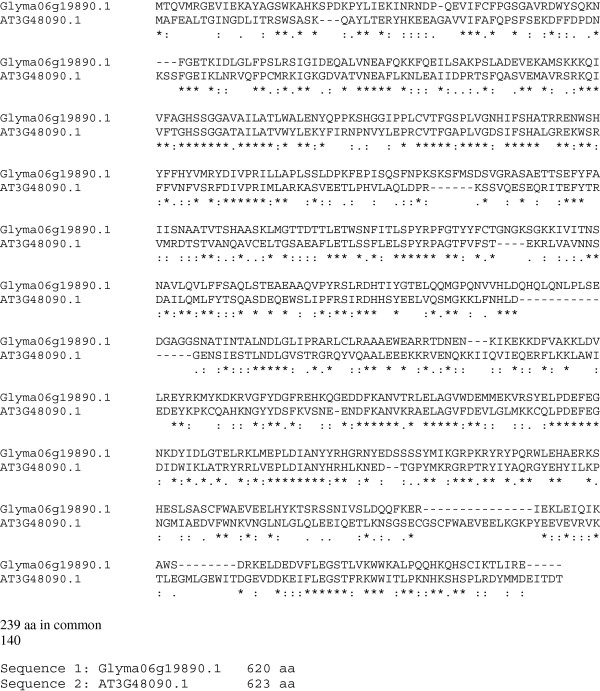
**Multiple sequence alignment of the *Arabidopsis *and soybean protein sequences of EDS1 using Clustal 2.1.** (*) = identical aa; (:) = highly conserved aa substitution; (.) = conserved substitution.

PAD4 is found upstream of SA production ([113]; Figure 1). PAD4 is a lipase like protein [48] that can form molecular complexes with EDS1 to modulate SA defense signaling [114]. Ectopic expression of *PAD4* reduces feeding time of green peach aphids on transgenic *Arabidopsis* plants. The aphid spends more time actively feeding on *pad4* mutants [115]. Previously, we showed that *AtPAD4* conferred resistance to SCN and RKN [60]. EDS1 is also upstream of SA, and it can interact with PAD4 [114,116,117]. Recently, Pant et al. [103] demonstrated that *GmEDS1* (Glyma06g19890) had a great effect on SCN and reduced SCN cysts by approximately 80%. This is in contrast to our data indicating that overexpression of *AtEDS1* did not decrease the FI of SCN. GmEDS1 is composed of 620 aa and AtEDS1 is composed of 623 aa (Figure 10). The amino acid sequences of GmEDS1 and AtEDS1 have 239 aa in common. Furthermore, they have another 140 aa that are closely related substitutions. Apparently, this conservation is not enough for AtEDS1 to provide resistance to SCN as did GmEDS1.

The *EDS5* gene, also found upstream of SA, encodes a membrane protein with homology to multidrug and toxin extrusion (MATE) transporters [50]. The *eds5* mutant accumulates very little SA and exhibits a reduction in PR-1 transcripts when infected with nematodes [19,118]. We show that overexpression of *AtEDS5* reduced the number of mature female cysts only modestly, to 75% of the control.

ACBP3 is one of six acyl-coenzyme A (CoA)-binding proteins in *Arabidopsis*[66]. ACBP binds to acyl-CoA esters and protects acyl-CoAs from degradation [119]. Bovine ACBP overexpression in yeast leads to an increase in the acyl-CoA pool size [120]. Overexpression of *AtACBP6* increased freezing tolerance in Arabidopsis [121]. These plants also showed a decrease in phosphatidyl choline and an increase in phosphatidic acid. Infection of *Arabidopsis* by either *Botrytis cinerea* or *P. syringae* pv *tomato* DC3000 induces the expression of *AtACBP3*, as does treatment with the fungal elicitor arachidonic acid [75]. The authors also showed that resistance to *P. syringe* was conferred by *ACBP3* overexpression in an NPR1-dependent manner and that *PR-1*, *PR-2,* and *PR-5* were constitutively expressed. When we overexpressed *AtACBP* in transgenic soybean roots, the number of SCN cysts decreased to 53% of the control at 35 dai.

The ACD2 gene in *Arabidopsis* encodes red chlorophyll reductase [122], which catalyzes the degradation of the porphyrin portion of chlorophyll [123]. ACD2 modulates cell death in Arabidopsis infected with *P. syringae.* It is localized to the chloroplast. Upon infection by *P. syringae,* localization of the protein changes, and it is localized in chloroplasts, mitochondria, and to a lesser degree the cytosol [84]. The accumulation of chlorophyll breakdown products may trigger cell death [122]. When we overexpressed the *AtACD2* gene in soybean roots, the FI of SCN was reduced to 55% of the control.

### Jasmonic acid

JA and ethylene are also important in the plant defense response, especially the response to necrotrophic pathogens [58]. JA and ethylene interact antagonistically with SA. Mechanical damage and wounding activates JA synthesis in Arabidopsis, potato, tomato, and other plants [58,124,125]. The role of JA in defense against nematodes is being examined by several laboratories. There are recent reports that JA is involved in defense in rice plants against nematodes [126,127]. Exogenous application of methyl-JA on rice shoots reduced galls by 63% per plant. In contrast, Bhattarai et al. [128] showed that JA is not required for resistance in tomato to RKN. They used nearly isogenic tomato cultivars resistant and susceptible to RKN to study gene expression using microarrays. The tomato *jai1* mutant, altered in JA signaling, reduced the susceptibility of tomato to RKN. Furthermore, they showed that auxin-related genes were differentially expressed in compatible and incompatible interactions with RKN. Neither foliar spray nor soil-drenching of tomato plants with SA, JA, or methyl-JA affected galling of roots by RKN [129]. We show that overexpression of three genes involved in JA/JA_ile_ production, *AtAOS, AtAOC,* and *AtJAR1*reduced the FI of SCN to 66% (P = 0.06) , 75% (P = 0.6) and 69% (P = 0.06) of the control. Although, the data are at the borderline of significance, the trend suggests that JA/ JA_ile_ may provide some degree of resistance to SCN in soybean. Further work is needed in this area, as the effects of SA and JA in roots has not been explored. Nor has SA-JA antagonism been documented in root systems. Perhaps, SA and JA interactions are not completely antagonistic at all times in all tissues. Or perhaps, exogenous application of plant hormones can provide resistance to nematodes, but the level of the plant hormone necessary to achieve resistance is not normally achieved during nematode attack.

### Genes increasing susceptibility of soybean to SCN

Overexpression of *AtDND1* resulted in the greatest increase in susceptibility of soybean roots to SCN of all genes tested here. DND1 is a known negative regulator of plant immunity [130-132]. Its promoter is the target of the transcriptional co-repressor, Topless-related 1 (TPR1), which may function through repression of negative regulators to activate R protein-mediated immunity responses [133]. When *AtDND1* was overexpressed in soybean roots, it decreased resistance to SCN as reflected by the female Index of 175% as compared to the control.

### *Arabidopsis* defense genes not impacting SCN susceptibility

Overexpression of *AtCDR1*, which encodes an apoplastic aspartic protease, resulted in a non-significant increase in susceptibility to SCN. This result contrasts with those of previous studies on plant resistance to bacterial and fungal pathogens. Overexpression of *CDR1* in T-DNA activation tagging studies yielded dwarf *Arabidopsis* plants and increased resistance to *P. syringae*[134]. Antisense *CDR1 Arabidopsis* plants were compromised in resistance to *P. syringae.* A rice aspartic protease, encoded by *OsCDR1*, was identified by Presad et al. [135]. When they overexpressed *OsCDR1* in *Arabidopsis,* the plants were more resistant to the necrotrophic fungal pathogen *Alternaria brassicicola*. When the gene was overexpressed in rice plants, the plants were more resistant to *Xanthomonas oryzae,* the rice blast fungus, and to *Magnaporthe oryzae,* which causes bacterial blight*.*

## Conclusions

Expression of several *Arabidopsis* genes provided protection to soybean against SCN. In fact, several genes provided increases in resistance comparable to or better than that provided by *Rhg1* and *Rhg4*, two naturally occurring resistance gene loci in soybean. However, not all *Arabidopsis* genes provided resistance. In fact, overexpression of *AtEDS1* did not increase resistance, although a *GmEDS1* ortholog was recently reported as providing resistance. These data indicate that some *Arabidopsis* genes can be used directly in soybean to confer resistance, especially genes associated with SA regulation, signaling, and synthesis, but not all *Arabidopsis* orthologs will provide the same results as the orthologous soybean gene. These and similar studies may provide useful insights into protein conservation and function, and several of these *Arabidopsis* genes may prove useful in engineering plants with broad resistance to nematodes.

## Methods

### Bioinformatics

Thirty-one genes were selected from published studies defining *Arabidopsis* mutants displaying phenotypes affecting SA and JA production, regulation, and signaling. The DNA sequence of the gene was obtained from The Arabidopsis Information Resource (TAIR; http://www.arabidopsis.org/). The DNA sequences of soybean genes used in multiple sequence alignments with *Arabidopsis* genes were obtained at Phytozome.net (Joint Genome Institute, U.S.D.O.E., Center for Integrative Genomics, U.C. Berkeley) using the *Glycine max* genome [136]. Primers for PCR amplification of the open reading frame of each gene were designed using Primer 3 (http://biotools.umassmed.edu/bioapps/primer3_www.cgi) or OligoAnalyzer 3.1 (Integrated DNA Technologies, Coralville, IA.) Multiple sequence alignments were made using CLUSTAL 2.1 (http://www.genome.jp/tools/clustalw/). Protein domains were identified using Pfam (http://pfam.sanger.ac.uk/search).

### Amplification and cloning of ORFs

The open reading frames (ORFs) of *Arabidopsis* target genes (Additional file 1: Table S1) were amplified by PCR and cloned into pRAP15 using the Gateway® system (Invitrogen, Carlsbad, CA) as described previously [8,60]. Templates for PCR were from cDNA libraries derived from *Arabidopsis* RNA. *Arabidopsis* cDNA was constructed from RNA extracted from *A. thaliana* (Columbia) whole plants and converted into cDNA as described by [8,60]. ORFs were PCR amplified using gene-specific PCR primers that contained CACC at the 5′end of the forward primer for directional cloning using the Gateway® (Invitrogen) system (Additional file 1: Table S1).

The PCR-amplified ORFs were cloned into pENTR using a pENTR™ Directional TOPO® Cloning Kit (Invitrogen) and transformed into *Escherichia coli* using One Shot® Mach1™ T-1 chemically competent cells (Invitrogen). Transformed colonies were selected using 50 μg mL^−1^ kanamycin. Each cloned ORF was DNA sequenced using the vector-specific primers M13-F and M13-R to confirm identity and integrity (Additional file 3: Table S3). Then, each ORF was directionally cloned into pRAP15, a gene expression vector [8,9], at the attR1 and attR2 sites using Invitrogen’s Gateway® technology and LR Clonase™ II Enzyme Mix (Invitrogen). The Clonase II reaction product was used to transform *E. coli* cells, and transformed colonies were selected on 10 μg mL^−1^ tetracycline plates. Presence of the insert in the correct orientation downstream from the FMV promoter was confirmed by PCR using the FMV-specific primer FMV-F (Additional file 3: Table S3) and the *A. thaliana* gene-specific reverse primer. The pRAP15 vector bearing each ORF was used to transform competent *Agrobacterium rhizogenes* ‘K599’ cells using the freeze-thaw method [137] with selection on 5 μg mL^−1^ tetracycline plates. Presence of the ORF in the pRAP15 vector was confirmed as described above. Expression of the ORF was controlled by the Figwort Mosaic Virus (FMV) promoter. The pRAP15 vector contains the gene encoding enhanced green fluorescent protein gene (eGFP) [138] regulated by the *rolD* promoter to provide strong eGFP expression in the root for identification of transformed roots. Presence of the gene encoding eGFP was confirmed by PCR using eGFP-F and eGFP-R primers (Additional file 3: Table S3), and eGFP was confirmed visually in transgenic roots. Presence of the *A. rhizogenes* R_i_ plasmid was confirmed by PCR using R_i_-F and R_i_-R primers (Additional file 3: Table S3).

### Formation and confirmation of composite soybean plants

Composite soybean plants consisting of untransformed shoots and transformed roots were produced as described previously [139,140]*A. rhizogenes* clones containing each ORF were grown as described previously [8]. Transformed control roots were produced using *A. rhizogenes* containing empty pRAP15 with no ORF. Briefly, one hundred soybean cv. Williams 82 PI518671 plants were grown in Promix in the greenhouse for 5–7 days. The plantlets were cut at the soil line and transformed with *A. rhizogenes* grown to an OD_600_ of 0.5. The stems were rinsed, and the plantlets were planted in the greenhouse and grown for four to five weeks. The plantlets were gently removed from the Promix, and non-transformed roots were excised. Transformed roots were retained after being recognized by fluorescence of eGFP using a Dark Reader Spot lamp (Clare Chemical Research, Dolores, CO). Plants were replanted in Promix and grown an additional two weeks. The non-transformed roots were removed a second time and approximately 12 to 20 healthy plants with only transformed roots were planted in sand and inoculated with SCN.

The presence of Arabidopsis genes in soybean transgenic roots was confirmed by PCR. Briefly, transgenic soybean roots from each construct were harvested, flash-frozen in liquid nitrogen in 2 ml microfuge tubes, and stored at -80C. After grinding 100 mg root tissue with a mortar and pestle in liquid nitrogen, total RNA was extracted using the RNAeasy Plant Mini Kit (Qiagen, Valencia, CA). Extracted samples of RNA were treated with TURBO™ DNAse I (Ambion, Carlsbad, CA) to remove residual genomic DNA. RNA was tested for genomic DNA contamination by PCR amplification using soybean primers for the soybean gene AW31036. No amplification products were produced when the RNA samples were used as template, but an amplification product was produced when genomic DNA served as template. One milligram of each RNA was converted to cDNA using the Superscript III First-Strand Syntheses System for RTPCR (Invitrogen, Carlsbad, CA), using oligo (dT)_12–18_ to prime the first strand of cDNA. To test the presence of the Arabidopsis constructs in the transgenic soybean roots, each cDNA served as template for amplification using gene-specific primers in a PCR reaction with Taq DNA polymerase (Invitrogen). Ten microliters of each reaction was electrophoresed on a 2% SB agarose gel for one hour at 150 volts. A 1Kb Plus ladder (Invitrogen) was included to estimate the size of the amplicons. The gel was photographed using a UV light box with an EOS Rebel T3i camera (Canon, Arlington, VA) with a HD UV filter (Canon). Images created with EOS imaging software (Canon) were annotated in Adobe Imaging software (Adobe, San Jose, CA). The nine gene-specific primers were tested by PCR to confirm that Arabidopsis cDNA was the source of the amplicon and not soybean DNA. PCR containing Arabidopsis cDNA produced amplicons, and only the positive control soybean-derived primer pairs gave amplification products when soybean DNA was used, confirming that the primers were specific to only the Arabidopsis genes within the soybean roots.

### Preparation of nematodes

SCN line NL1-RHg was maintained on susceptible *Glycine max* cv. ‘Essex” as described previously [141]. Roots were washed to dislodge SCN cysts, which were captured between nested 850-μm and 250-μm sieves. Cysts were purified by sucrose flotation [142] and crushed against a 7.6-cm diameter 250-μm sieve with a rubber stopper partially submerged in water to release the eggs. Eggs captured in a tray below the sieve were poured through a 61-μm sieve and collected on a 25-μm sieve. Eggs were cleaned by soaking in 0.5% sodium hypochlorite for 1.5 minutes, and then rinsed in sterile deionized distilled water. The eggs were poured into a small tray and hatched in a solution of 3 mM ZnSO_4_ on a rotary shaker at 26°C and 25 rpm. After four days, the hatching solution was passed through a 30-μm mesh nylon cloth (Spectrum Labs Inc, Rancho Dominguez, CA), which retained the unhatched eggs and liberated the J2 stage SCN in the solution collected below the cloth. To concentrate the J2s, 200 mL of the solution was placed in a 1 L glass beaker and placed on a rotary shaker at 100 rpm. J2s were collected from the center bottom of the beaker with a Pasteur pipette. Three 5-μL aliquots of the J2 solution were examined under the microscope to determine the concentration and viability of the J2s. The solution was diluted to a concentration of 1000 J2 mL^−1^ with sterile water.

### Nematode assay

Twelve transformed composite plants for each construct tested were inoculated with 2000 J2 nematodes per plant. Two holes 4-cm deep were made in the sand on either side of each plant. One mL of a 1000 J2 mL^−1^ suspension was added to each hole and covered with sand. At 35 days after inoculation (dai), the cysts were collected from the roots of each plant between nested 850-μm and 250-μm sieves and rinsed onto lined filter paper in a Buchner funnel under vacuum [143]. Cysts were counted under a dissection microscope. Plant roots transformed with empty vector were used as the positive control for the female index as described below.

### PCR assays

Expression of ORFs in transformed soybean roots was confirmed by RT-PCR as described previously [60]. Three individual soybean roots were harvested per construct. RNA was extracted using a Qiagen RNeasy Mini Kit according to the manufacturer’s instructions. Contaminating DNA was removed by DNase digestion using a TURBO™ DNase kit (Ambion) according to the manufacturer’s instructions. RNA was tested as template by PCR to confirm that no contaminating DNA was present. The RNA was converted into cDNA using the Superscript III First-Strand Synthesis System for RT-PCR (Invitrogen) according to manufacturer’s instructions. Soybean roots transformed with pRAP15 served as controls. Primers were designed to produce an amplicon between 100 and 200 bp (Additional file 2: Table S2). The gene encoding rs-21 (Glyma09g00210.1) served as a positive control [144]. Reactions containing no RNA were used as negative controls.

RT-PCR reactions were conducted in triplicate for each root sample using the Brilliant II SYBR® Green QPCR Master Mix Kit (Agilent Technologies) according to the manufacturer’s instructions. Primer sequences for RT-PCR are provided in Additional file 2: Table S2. Genomic DNA (gDNA) was isolated from individual roots using the DNeasy Plant Mini kit (Qiagene, USA). Three independently transformed roots were examined for each gene transformation. The gDNA served as template in PCR containing primers specific to the *Arabidopsis* gene.

### Quantitative real-time polymerase chain reaction (qRT-PCR)

Three individual roots (100 mg each) were collected that were transformed with either *AtNPR1*, *AtTGA2*, or the empty pRAP15 vector as control, respectively. Each root represented an independent transformation event. RNA was extracted from each root using an Ultra Clean Plant RNA Isolation Kit (MOBIO, Carlsbad, CA). Genomic DNA was removed using DNase I. Single-stranded cDNA was synthesize from the RNA using SuperScript III First-Strand Synthesis System (Invitrogen, Carlsbad, CA) and oligo dT primers, according to the manufacturer’s instructions. All qRT-PCR primer pairs were designed to flank a region that contains one intron to ensure that product was amplified from cDNA. Primers (Additional file 4: Table S4) were specific to the flanking region of the Arabidopsis *AtNPR1* and *AtTGA2* genes, yielding amplicons of approximately 150 bp. The soybean ubiquitin-3 (*GmUBI-3*) gene, GenBank accession D28123, served as a positive qRT-PCR control to demonstrate that soybean RNA was present in all samples. Expression levels of the defense-related soybean genes *ERF1* (Glyma20g34570), encoding the ethylene response factor 1; *CHIB1* (Glyma10g27870), encoding a basic chitinase protein; and *PR-5* (Glyma05g38110), encoding an osmotin-like protein also were determined by qRT-PCR. Other controls for qRT-PCR included reactions containing no template and qRT-PCR reactions containing no reverse transcriptase. qRT-PCR was performed on three biological replicates with each reaction replicated three times. The Stratagene Mx3000P Real-Time PCR system (Stratagene, La Jolla, CA) was used to determine transcript abundance as described by the manufacturer. SYBR Green was used to measure DNA accumulation during the reaction. The Ct (cycle at which there is the first clearly detectable increases in fluorescence) values were calculated using software supplied with the Stratagene Mx3000P Real-Time PCR system. The dissociation curve of amplified products was used to demonstrate the production of only one product per reaction. To further ensure that only one product was formed in each reaction, the PCR products were analyzed on 0.8% agarose gels and visualized under UV light. Absolute quantification of transcript levels was performed according to the sigmoidal model described by (Rutledge and Stewart, 2008) [62].

### Statistical analysis

Outliers in the female count data were removed using Grubbs’ test [145] at the GraphPad QuickCalcs Web site (http://graphpad.com/quickcalcs/grubbs1/). Normality of the data was checked using the Shapiro-Wilk test ([146]; online version implemented by S. Dittami, http://scistatcalc.blogspot.com/2013/10/shapiro-wilk-testcalculator.html). Means were compared using Welch’s unpaired *t* test for unequal variance [147,148] at the GraphPad QuickCalcs Web site (http://graphpad.com/quickcalcs/ttest1/). The female index (FI) was calculated as follows: FI = (N_g_/N_c_) X 100, where N_g_ = mean number of females for the gene of interest and N_c_ = mean number of females for the empty pRAP15 control.

The female index was calculated as described below from 7–16 experimental and 10 or more control plants [8,60,61,139,140,149-153]. Experiments on Arabidopsis genes overexpressed in roots of soybean composite plants were conducted according to published procedures, such as those of Golden et al. [153], Riggs and Schmidtt [149,150] Kim et al. [151] and Niblack et al. [152]. In the experiments of Golden et al. [153], the labs that originally developed and modified the FI, the FI is typically calculated from a total of 3–10 experimental and 3–10 control plants, each individual plant serving as a replicate. Experimental replicates may or may not be performed. All of the experiments presented here exceed these published studies in that regard and are conducted with similar plant numbers to recently published studies [8,9,60,61,139,140,154]. In the present analysis, the number of experimental plants met or exceeded that in investigations testing SCN infection in genetically engineered soybean [155-158]. Herein, we also report standard error of the mean (SEM) for experimental and control groups.

### Availability of supporting data

The data supporting the results of this article are included within the article.

## Abbreviations

AOC: Allene oxide cyclase; AOS: Allene oxide synthase; CM: Chorismate mutase; dai: days after inoculation; ET: Ethylene; FI: Female index; gDNA: genomic DNA; IAA: Indole acetic acid; ICS: Isochorismate mutase; JA: Jasmonic acid; LOX: Lipoxygenase; OPR3: 12-oxophytodienoic acid reductase; ORFs: Open reading frames; PAL: Phenylalanine ammonia lyase: PAMP, Pathogen-associated molecular patterns; PCR: Polymerase chain reaction; PR: Pathogenesis-related; RKN: Root-knot nematode; SA: Salicylic acid; SAR: Systemic acquired resistance; SCN: Soybean cyst nematode.

## Competing interests

The authors have no competing interests.

## Authors’ contributions

BM conceived of and designed the experiments, analyzed the data, drafted the manuscript; HB grew the plants, transformed the plant roots, and trimmed the roots; MM inoculated roots with nematodes, harvested and counted nematodes; SK trimmed the roots, harvested and counted the nematodes, and cloned genes; EB cloned genes, transformed the plant roots, and trimmed the roots; RY cloned the genes, trimmed the roots, harvested and counted the nematodes. All authors read and approved the final manuscript.

## Supplementary Material

Additional file 1: Table S1 Primers used to PCR amplify ORFs for cloning into pRAP15. Click here for file

Additional file 2: Table S2 Primers used in RT-PCR assays. Click here for file

Additional file 3: Table S3 Primers used to confirm clone identity. Click here for file

Additional file 4: Table S4 Primers used for qRT-PCR. Click here for file
