# Supplementary material for: Arabidopsis genes, AtNPR1, AtTGA2 and AtPR-5, confer partial resistance to soybean cyst nematode (Heterodera glycines) when overexpressed in transgenic soybean roots
Source: BMC Plant Biol. 2014 Apr 16;14:96. doi: 10.1186/1471-2229-14-96 (PMC4021311; doi:10.1186/1471-2229-14-96)
Supplement: Additional file 1: Table S1 — Primers used to PCR amplify ORFs for cloning into pRAP15. [file 1471-2229-14-96-S1.doc]

Additiona file 1: Table S1. Primers used to PCR amplify ORFs for cloning into pRAP15.

| **Phytozome ID** | **Gene** | **Forward primer** | **Reverse Primer** |
| --- | --- | --- | --- |
| AT1G64280.1 | NPR1 | CACCTTGGCTCTGCTCGTCAA | TTACACTAAGAGGCAAGAGTC |
| AT5G06950.1 | TGA2 | CACCGTACAGAAAAGTGGTGA | TGAATTACCACAACCATGTAG |
| AT1G75040.1 | PR-5 | CACCAAATATGGCAAATATCTCCAGTATTC | TTAAGGGCAGAAAGTGATTTCGTAG |
| AT4G24230.6 | ACBP3 | CACCATTGACGGACCCGCTTCC | ATCCCAAAGTCCTCCTTCAAGCC |
| At4G37000.1 | ACD2 | CACCAAAAAATGGCGATGATATT | GGTCATCTTGCGTTCTTGTG |
| AT1G69370.1 | CM-3 | CACCTCTCCGATGGAGGCTAAGTTAC | TTAATCCAGTCTTCTAAGCAAGTACTC |
| AT5G42650.1 | AOS | CACCACTCGCCACTGTTTCG | ACAGATGGACTACACAGGTGC |
| AT5G50260.1 | CEP1 | CACCACTCAATCACAATCACAA | ACGATTCCCATCAACTTCATAC |
| AT1G74710.2 | ICS1 | CACCGAATTTCTGCAATGGCTTC | GTTACAACCCGAAAAGGCTCG |
| AT2G46370.4 | JAR1 | CACCATCCGTTTCGTCTGATC | GGTAACGTCACATCAGAAAT |
| AT3G03600.1 | RPS2 | CACCTTCCGACGACGAACT | CTGAAGAACAATTATTCCAGACTCAG |
| AT5G48485.1 | DIR1 | CACCGTGACTATATGGAGCAATCCA | CGAAATTGCCCTTCCGTGTTTGG |
| AT4G39030.1 | EDS5 | CACCATGTAATTTCGCAGAAGAGA | TGTGAAGAGTCGATCTATCAGTG |
| AT3G25760.1 | AOC | CACCACCAAAGTCCCATCTCT | ATACAGGACACGAGAAAGATAAGAC |
| AT3G26830.1 | PAD3 | CACCCAAGGAAAATGTCGGT | ATTCAGTGGTGAAGAACTTGAA |
| AT5G54250.1 | DND2 | CACCCATGCGTTAGTACTTTCA | CCACATGAACTATGAGAAGAGG |
| AT3G20600.1 | NDR1 | CACCTACCAAATTCTTGAAAAC | CTAAGGATCACAACAAAACTC |
| AT2G14610.1 | PR-1 | CACCTCTCTATATAAGCGATGTTTACGA | GATACATCCTGCATATGATGCTCC |
| AT5G13160.1 | PBS1 | CACCCATTGATTCCCCAGTAG | GG TCA AGA CCG ACC CAA ACC |
| AT1G02170.1 | LOL3 | CACCATCCGATTCGTCTTCATCTG | GATTCTCAAATCACACTCGCTATACAT |
| AT3G25070.1 | RIN4 | CACCTCAGACTTCAATTCTTTAGC | TCATCAAGATTACACACTGAT |
| AT4G20380.8 | LSD1 | CACCACCCTTCTCCAATCGAAG | CAAACCAAACGCAGGAATAGAGG |
| AT4G11260.1 | SGT1b | CACCTCTCATGGCTTCCGATCTC | GCAACACTGATCCAGAAAATGTC |
| AT5G64930.1 | CPR5 | CACCATAAGTTATATTTTAAGGCTATGGAAGC | CTCCTCGTCTCAAGCATAGTC |
| AT1G12560.1 | ExPA7 | CACCAAGAGGCTAGAATGGG | AGGAGAAGAGAAAACTCCGT |
| AT3G48090.1 | EDS1 | CACCATGTAATTTCGCAGAAGAGA | TGTGAAGAGTCGATCTATCAGTG |
| AT2G17265.1 | DMR1 | CACCAGGCTTCGTCTTTGTTC | CCAATCTTAACATAATCAAACAGCACAC |
| AT1G05180.1 | AXR1 | CACCGTCTCTCGCTTGAGC | GATTCTGAGTCGGATGGTCTCA |
| AT4G21610.1 | MC2 | CACCAGACGGAAGGCGTG | AATTGACAAAAATCGGTTCAAGT |
| AT5G33340.1 | CDR1 | CACCACGTCAGATTCTCTGAACAC | CAAGGAAATACAAAGCATCACC |
| AT5G15410.1 | DND1 | CACCTCTATTTCAATCATGCCCTCTCAC | TTGAGGAGGAACAATGAACACAC |
