# Supplementary material for: Arabidopsis genes, AtNPR1, AtTGA2 and AtPR-5, confer partial resistance to soybean cyst nematode (Heterodera glycines) when overexpressed in transgenic soybean roots
Source: BMC Plant Biol. 2014 Apr 16;14:96. doi: 10.1186/1471-2229-14-96 (PMC4021311; doi:10.1186/1471-2229-14-96)
Supplement: Additional file 2: Table S2 — Primers used in RT-PCR assays. [file 1471-2229-14-96-S2.doc]

Additional file 2: Table S2. Primers used in RT-PCR assays.

| **Gene** | **Forward primer** | **Reverse primer** | **Amplicon**  **Size (bp)** |
| --- | --- | --- | --- |
| **NPR1** | CACTATGGCGGTTGAATGTA | GGGAGGAACATCTCTAGGAA | 124 |
| **TGA2** | TTATCGAGCGGGACTCTT | CCTTCGAGTGTACCTAACTTTC | 98 |
| **PR5** | AGCCTCGTAGATGGTTACA | GACACAGCCTGCGTATTT | 81 |
| **ACBP3** | AGTGGTGGAGATGTCTTAGT | CCTCTCAATTCCTTCCCAATC | 150 |
| **ACD2** | TGACCTCCCACATCGTAAA | CAGAACGGACAAAGAGAGAAG | 149 |
| **CM3** | CTGCTCTCTGTGACACAAT | TCACGAAACTTGGCTTCA | 85 |
| **MC2** | GGCGTTTGCTTTCTTATCT | CCAACCTGGTTAGCTTCA | 91 |
| **CDR1** | CGTCTCAAGAGACCTTCTATTAC | CTGAGCCTGAGTATTGGATTT | 78 |
| **DND1** | GGTTCTAAGTGGCTTGTTACT | CACCATTCCATATCCCTACAC | 117 |
| **AW310136** | CTGTGCTCATGCAGAGGAAT | CTCGTGGAGTTTGGTCTCAA | 134 |
