# Supplementary material for: Arabidopsis genes, AtNPR1, AtTGA2 and AtPR-5, confer partial resistance to soybean cyst nematode (Heterodera glycines) when overexpressed in transgenic soybean roots
Source: BMC Plant Biol. 2014 Apr 16;14:96. doi: 10.1186/1471-2229-14-96 (PMC4021311; doi:10.1186/1471-2229-14-96)
Supplement: Additional file 3: Table S3 — Primers used to confirm clone identity. [file 1471-2229-14-96-S3.doc]

Additional file 3: Table S3. Primers used to confirm clone identity

| **Primer ID** | **Primer Sequence** |
| --- | --- |
| **M13F** | CGTTGTAAAACGACGGCCAG |
| **M13R** | CTGCCAGGAAACAGCTATGAC |
| **Intron2R** | CCAGTTAACGTGTCTCATAT |
| **FMVF** | AAGAAGCCCTCCAGCTTCAAAG |
| **eGFPF** | ATGGTGAGCAAGGGCGAGGAGC |
| **eGFPR** | TCGTCCATGCCGAGAGTGATCCCG |
| **RiF** | TCAGCCTCCCCGCCGGATG |
| **RiR** | ATGCAAAAGACAGGATTGATCGCA |
