# Supplementary material for: Arabidopsis genes, AtNPR1, AtTGA2 and AtPR-5, confer partial resistance to soybean cyst nematode (Heterodera glycines) when overexpressed in transgenic soybean roots
Source: BMC Plant Biol. 2014 Apr 16;14:96. doi: 10.1186/1471-2229-14-96 (PMC4021311; doi:10.1186/1471-2229-14-96)
Supplement: Additional file 4: Table S4 — Primers used for qRT-PCR. [file 1471-2229-14-96-S4.doc]

Additional file 4: Table S4. Primers used for qRT-PCR

| **Gene primer** | **Primer Sequence** |
| --- | --- |
| ***AtNPR1-R*** | GGGAGGAACATCTCTAGGAA |
| ***AtTGA2-F*** | TTATCGAGCGGGACTCTT |
| ***AtTGA2-R*** | CCTTCGAGTGTACCTAACTTTC |
| ***Ubiquitin-3-F*** | GTGTAATGTTGGATGTGTTCCC |
| ***Ubiquitin-3-R*** | ACACAATTGAGTTCAACACAAACCG |
| ***GmPR5-F*** | CCCAATAACCTCTTCTATTACGG |
| ***GmPR5-R*** | CACGTTTCATAGCATATGACCAT |
| ***GmCHIB-F*** | GAG GGC CAA TTC AAC TTT CA |
| ***GmCHIB-R*** | TGC TGT CAT CCA AAA CCA GA |
| ***GmERF1-F*** | GGGAAGGGGATGCACACAACCAAGG |
| ***GmERF1-R*** | GTTGGCCATTCCATCCTTCCACCACCT |
